# Knowledge and attitudes of nursing students regarding the sexuality of older adults: a quasi-experimental study

**DOI:** 10.1590/0034-7167-2024-0011

**Published:** 2024-12-13

**Authors:** Ana Carolina Macri Gaspar Vendramini, Rosemeiry Capriata de Souza Azevedo, Priscila Aguiar Mendes, Janderson Diego Pimenta da Silva, Annelita Almeida Oliveira Reiners, Amanda Cristina de Souza Andrade

**Affiliations:** I Universidade do Estado de Mato Grosso. Tangará da Serra, Mato Grosso, Brazil; II Universidade Federal de Mato Grosso. Cuiabá, Mato Grosso, Brazil; III Universidade Federal de Minas Gerais. Belo Horizonte, Minas Gerais, Brazil

**Keywords:** Aged, Sexuality, Sexual Behavior, Students, Nursing, Sexually Transmitted Diseases, Anciano, Sexualidad, Conducta Sexual, Estudiantes de Enfermería, Enfermedades de Transmisión Sexual

## Abstract

**Objectives:**

to compare the knowledge and attitudes of nursing students regarding sexual behavior and sexually transmitted infections (STIs) in older adults before and after an educational intervention.

**Methods:**

this quasi-experimental study involved a convenience sample of 45 nursing students from a public university, conducted in three stages: pre-intervention, intervention, and post-intervention. A questionnaire was used to assess sociodemographic characteristics, academic training, and knowledge and attitudes on the topic. The intervention was an educational web conference. Paired t-test and Wilcoxon test were used for data analysis.

**Results:**

there was a statistically significant difference in the knowledge and attitude scores of nursing students before and after the educational intervention (p < 0.001). A significant increase was observed in the knowledge score (from 9.3 to 12.2) and attitude score (from 108 to 117.2) in the post-intervention phase.

**Conclusions:**

the knowledge of nursing students regarding the sexuality of older adults increased after the educational intervention, and their attitudes on the subject became more positive.

## RESUMEN

**Objetivos:**

comparar el conocimiento y las actitudes de los estudiantes de enfermería sobre el comportamiento sexual y las Infecciones de Transmisión Sexual en personas mayores antes y después de una intervención educativa.

**Métodos:**

estudio cuasi-experimental, con una muestra de conveniencia de 45 estudiantes de enfermería de una universidad pública, realizado en tres etapas: pre-intervención, intervención y post-intervención. Se utilizó un cuestionario sobre características sociodemográficas, formación académica y conocimiento y actitudes sobre el tema. La intervención fue educativa a través de una conferencia web. Se utilizaron la prueba t pareada y la prueba de Wilcoxon para el análisis de los datos.

**Resultados:**

hubo una diferencia estadísticamente significativa en los puntajes de conocimiento y actitudes de los estudiantes de enfermería antes y después de la intervención educativa (p < 0,001). Se observó un aumento significativo en el puntaje de conocimiento (9,3 a 12,2) y actitud (108 a 117,2) en la post-intervención.

**Conclusiones:**

el conocimiento de los estudiantes de enfermería sobre la sexualidad de las personas mayores aumentó después de la intervención educativa y sus actitudes respecto al tema se volvieron más positivas.

## INTRODUCTION

One of the significant challenges today is that, in recent years, various countries, including Brazil, have seen a considerable increase in cases of Sexually Transmitted Infections (STIs) among older adults^(1-4)^. Epidemiological data show that in Japan, a high-income country, for example, syphilis cases increased approximately eightfold, from 165 in 2009 to 1,280 in 2019, with a 28.7% increase in men and a 23.1% increase in women in the incidence rate among older adults ^(1)^. In Brazil, a middle-high income country, there was a trend of stability regarding HIV, but with an increase in incidence among elderly people aged 60 and over, rising from 4.25/100,000 inhabitants in 2007 to 8.73/100,000 inhabitants in 2020, in the northeast region^(2)^.

Several factors related to the sexual behavior of older adults have contributed to their vulnerability to STIs, such as low risk perception for contracting STIs^(5)^ and unprotected sexual practices^(4,6)^. Additionally, they have little knowledge about STI and AIDS prevention methods^(5,6)^ and do not seek health units for STI/HIV testing^(7)^, contributing to a possible late HIV diagnosis.

Other factors are related to healthcare professionals, who, according to the literature, have difficulty addressing the topic of sexuality during the care of the elderly population^(8,9)^. Most professionals, including nurses, hold stigmas and stereotypes regarding the sexuality of older adults, believing that there is no sexual interest or active sexual life at this stage of life^(9-11)^. They feel embarrassed to discuss the subject and have negative attitudes towards the sexual activity or desire of this population^(12)^.

Moreover, they have little knowledge about HIV/AIDS in older adults^(7)^ and their sexual behavior^(10)^. One reason for this is that the academic training of these healthcare professionals is insufficient and/or non-existent regarding content on sexuality in aging^(11,13-15)^.

In nursing, few studies have investigated the knowledge and attitudes of students about sexuality, sexual behavior, or STIs specific to the elderly population. However, existing studies point to unsatisfactory knowledge on the subject, and attitudes oscillate between positive and negative^(16,17)^. Knowledge can be considered as what a person knows about a phenomenon. Attitude is a psychological reaction expressed by a positive or negative, favorable or unfavorable evaluation of an object, person, or event, and it has cognitive (information), affective (feelings), and behavioral (intentions to act) components^(18)^.

Research has shown that the knowledge level of students about sexual health can be improved with educational intervention programs, as well as promoting attitude changes^(7,19)^, but these studies are scarce. Moreover, none of them specifically address sexual health and STIs in older adults^(7,19)^. In this context, this research hypothesizes that an educational intervention can improve the knowledge and attitudes of nursing students about sexual behavior and STIs in older adults.

## OBJECTIVES

To compare the knowledge and attitudes of nursing students regarding sexual behavior and STIs in older adults before and after an educational intervention.

## METHODS

### Ethical aspects

The research was approved by the Research Ethics Committee, and registered in the Brazilian Registry of Clinical Trials (REBEC) under number RBR-3rkg96p.

### Study design, period, and location

The study was conducted from 2020 to 2021 at a public university in the state of Mato Grosso. It is a quasi-experimental, pre-and post-test study with an intervention group, guided by the CONSORT tool.

### Population, inclusion, and exclusion criteria

The study population consisted of 62 students enrolled in the nursing course at the university, who were in semesters seven through ten. This criterion was used because the course on elderly health is offered in the seventh semester. Those who did not attend classes despite being enrolled were excluded.

The sample was determined before data collection using a repeated measures sample calculation, considering a 5% significance level, 80% power, and a minimum difference of 2.5 units before and after the intervention, as per a study^(19)^, totaling 32 individuals. With an estimated 25% dropout rate, the minimum sample size was calculated to be 43 students. The following inclusion criteria were established: being a student aged 18 or older, of any gender.

All 62 enrolled students were invited to participate in the research. Of these, 54 accepted the invitation. Eight students discontinued the study as they did not complete all stages of the research: six did not participate in the first intervention meeting and two did not participate in the second. In the end, the sample consisted of 45 nursing students.

### Study protocol

**Figure 1:**
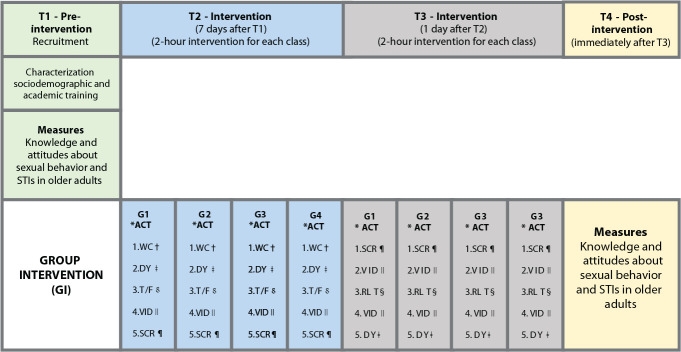
Representation of the data collection flowchart and intervention program

Data collection took place in November and December 2020, in three stages: pre-intervention, intervention, and post-intervention, as shown in Figure 1.

*ACT - Activity; †WC - Welcoming; ‡DY - Dynamic; §T/F - True/False; VID - Video; ¶SCR - Script; §RLT - Roulette.

In the pre-intervention phase (T1), nursing students were recruited. The invitation was made through WhatsApp^®^ group messages and emails containing an invitation letter with information about the research, a Free and Informed Consent Form (TCLE) signed by the researcher, and a link to the research questionnaire via the Google Forms^®^ platform.

In the second phase, the intervention, the 54 students were distributed into four groups according to their enrolled semester. Each group had two pre-scheduled meetings on consecutive days, lasting 2 hours each. Each meeting (T2 and T3) was planned with a specific theme. The first meeting was aimed at addressing activities related to the sexual behavior of older adults, and the second focused on STIs in this population.

The intervention was educational, conducted online (due to the COVID-19 pandemic) via web conference, using the Google Meet^®^ meeting application, and utilized workshop techniques, allowing a dialogic space in the teaching-learning process. The activities were developed by the researcher and accompanied by a tutor, who was responsible for organizing the activities and recording participant attendance. During all activities, participants were required to keep their cameras on.

In the first meeting (T2), participants were welcomed, socialized, informed about the activities to be developed, and attendance was checked. Subsequently, a dynamic activity was carried out to encourage students to express their ideas on the topic. For this, the method of free association of ideas and the interactive digital whiteboard tool were used, starting with the question: “What comes to mind when you hear the phrase ‘sexuality of older adults’?”. As students expressed their words, they were recorded on the board.

Next, a True or False dynamic was applied to improve knowledge and demystify myths about the sexual behavior of older adults. The researcher presented statements about the sexual behavior of older adults, and participants had to respond: true, false, or I don’t know; then, the correct answer was presented by the researcher based on scientific evidence.

Another activity was the presentation of a video about the sexuality of older adults, available on an online platform^(20)^, aimed at improving students’ attitudes toward the sexual behavior of older adults. At the end of the video, time was allocated for discussion about the information and perceptions gained from the video. At the end of this meeting, participants were asked to prepare a script, to be presented at the beginning of the next meeting, addressing the sexual behavior of older adults during a nursing consultation.

On the second day of the intervention (T3), the script previously prepared by the participants was discussed, and, in the end, a single instrument was constructed and shared with everyone. This activity aimed to address the approach to the sexuality of older adults, carried out by students during a nursing consultation.

Continuing, to improve the students’ knowledge about STIs, a video produced by the researcher was presented, containing epidemiological information about STIs and prevention policies for these diseases in Brazil and around the world. Next, to improve the students’ attitudes towards older adults with STIs, another video with testimonies from older adults living with HIV/AIDS, available on an online platform^(21)^, was shown. After each video presentation, there was a space for discussion on the topic among the participants.

Subsequently, to further improve the students’ attitudes towards older adults with STIs, a dynamic activity called “virtual roulette” was conducted by the researcher. This activity included numbers corresponding to questions prepared by the researcher about the actions of healthcare professionals related to the promotion of sexual health and STI prevention in older adults. Each student had to answer the question corresponding to the selected number on the roulette. After the participants’ responses, the researcher supplemented by presenting study results on the topic, opening spaces for reflection and group debate.

To conclude the educational activity, the researcher revisited the dynamic activity conducted in the first meeting, where the question about the sexuality of older adults was posed; then, the results of the two produced charts were presented to the students, who were encouraged to compare the changes that occurred after participating in the intervention. The researcher concluded the activity by providing an opportunity for students to evaluate the intervention.

Still, in the second meeting, the post-intervention phase (T4) was conducted. For this, the same instrument used in the pre-intervention phase to measure the knowledge and attitudes of nursing students about the sexual behavior and STIs of older adults was applied.

### Study Variables

The study variables were sociodemographic characteristics (sex, age group, marital status, religion, close contact with older adults, living with older adults); academic training (current semester, content on the sexual behavior of older adults, content on STIs in older adults, complementary activities on sexual behavior and STIs, research with older adults, extension activities with older adults, practical assistance with older adults); knowledge and attitudes about the sexual behavior and STIs in older adults (mean knowledge and attitude).

### Data Collection Instrument

Data were collected through a questionnaire to identify the characteristics of the study participants. It included closed-ended questions about the students’ sociodemographic and educational aspects. To identify knowledge and attitude, a scale created by the researchers on knowledge and attitudes about sexual behavior and STIs in older adults was used, which underwent a content validation process by a committee of eight judges with expertise in the area, achieving a content validity index (CVI) of 96%.

The knowledge scale consists of 13 questions with true, false, and I don’t know responses. For each correct answer, one point was awarded, and a total score between 0 and 13 points could be obtained. As for attitudes, the scale has 26 Likert-type questions, ranging from: strongly agree, partially agree, undecided, partially disagree, and strongly disagree. The final score ranges from 1 to 130 points. It was determined that the higher the score, the better the knowledge and the more positive the attitudes.

### Data Analysis and Statistics

The data were organized into a database using the Stata statistical program, version 16. To analyze the data, absolute and relative frequencies, mean, median, minimum, maximum, and standard deviation of the variables were calculated. To identify changes in knowledge and attitude, the results obtained from the instrument’s application before and after the intervention were evaluated. Initially, data normality was tested using the Shapiro-Wilk test. To compare the knowledge and attitude scores of nursing students about sexual behavior and STIs in older adults before and after the intervention, paired t-tests or Wilcoxon tests were used, with a significance level of 5%.

## RESULTS

Of the 45 nursing students, 93.3% are female, with an average age of 25 years, 60% are single, and 46.5% are Protestant. The majority (75.6%) do not live with people over 60 years old. Of those who reported (83.6%) having close contact with older adults, 35.6% have contact with grandparents, 22.5% with parents and/or uncles, and 22.5% with parents and/or grandparents.

Regarding academic training, 26.7% of the students are in the seventh and eighth semesters. During the course, most (55.6%) received some content on the sexual behavior of older adults; however, 53.3% did not receive significant content on STIs in this population. Respectively, 93.3% and 91.1% of the students did not attend lectures on sexual behavior or STIs in older adults. Slightly more than 60% of the students performed practical assistance with older adults without addressing aspects of sexual behavior or STIs. Concerning research and extension activities involving the elderly population, the majority did not participate in either activity (86.7% and 82.2%, respectively). Almost half of the students (44.4%) self-reported having little knowledge about the sexual behavior of older adults and STIs.

As shown in Table 1, the results related to the knowledge of nursing students about sexual behavior and STIs in older adults indicate a higher percentage of correct answers in the post-intervention phase compared to the pre-intervention phase. Notably, regarding sexual behavior, the questions about casual relationships, the use of sex toys and films to improve sexual performance, and the use of condoms stood out. Regarding STIs, the question about the available policies in Brazil for the prevention of STIs in the elderly population showed the greatest change in the percentage of correct answers.

**Table 1:** Distribution of nursing students according to items on the knowledge scale about sexual behavior and sexually transmitted infections in older adults before and after the intervention, Tangará da Serra, Mato Grosso, Brazil, 2020

Variables	Pre-intervention			Post-intervention		
	False n(%)	True n(%)	Don't know n(%)	False n(%)	True n(%)	Don't know n(%)
Older adults do not have an active sexual life	37(82.2%)	8 (17.8%)		42(93.3%)	3 (6.7%)	
The aging process causes physiological changes that affect the sexual performance of older adults	-	45(100%)	-	-	45(100%)	-
Sexual desire remains in old age	1(2.2%)	43(95.6%)	1(2.2%)	-	45(100%)	-
Older women evaluate their sexual satisfaction more positively than older men	23(51.1%)	8(17.8%)	14 (31.1%)	31(68.9%)	10(22.2%)	4(8.9%)
Casual sexual relationships are part of the sexual behavior of older adults	7(15.6%)	27(60%)	11(24.4%)	-	45(100%)	-
The frequency of sexual activity in older adults decreases with age	10(22.2%)	30(66.7%)	5(11.1%)	11(24.4%)	34(75.56%)	-
Sexual activity for older adults is not limited to intercourse; it can include kissing. hugging. caressing. and masturbation	-	43(95.6%)	2(4.4%)	-	45(100%)	-
Older adults use sex toys or films to improve sexual performance	2(4.4%)	20(44.4%)	23(51.2%)	-	44(97.8%)	1(2.2%)
Older adults use medications to improve sexual performance	-	41(91.1%)	4(8.9%)	-	45(100%)	-
Older adults often use condoms during sexual intercourse	30(66.7%)	4 (8.9%)	11(24.4%)	41(91.1%)	3 (6.7%)	1(2.2%)
There are many cases of HIV/AIDS. hepatitis. and syphilis in the older population	-	36(80.0%)	9 (20.0%)	-	45(100%)	-
The diagnosis of HIV/AIDS in older adults is often late	1(2.2%)	35 (77.8%)	9 (20.0%)	-	45(100%)	-
In Brazil. there is a policy for the prevention of STIs/HIV/AIDS in older adults	11(24.4%)	9(20.0%)	25(55.6%)	40(88.9%)	5(11.1%)	-

Regarding the attitudes of nursing students about the sexual behavior and STIs in older adults presented in Table 2, changes can be observed in all components of attitude: cognitive, affective, and behavioral, after the intervention. The percentage of correct responses from students in the post-intervention phase was higher for all questions except one (feeling embarrassed to teach older adults how to use a condom).

**Table 2:** Distribution of nursing students according to the attitude scale about sexual behavior and sexually transmitted infections in older adults before and after the intervention, Tangará da Serra, Mato Grosso, Brazil, 2020

Variables	Pre-intervention Correct answer n(%)	Post-intervention Correct answer n(%)
Cognitive Component		
I consider it important to assess the sexual behavior of older adults in nursing consultations ^*^	44 (97.8%)	45 (100.0%)
I believe that most older adults lose interest in sex †	02 (4.4%)	27(60.0%)
Healthcare professionals expect older adults to ask about sexual practices and STIs †	05 (11.1%)	12 (26.7%)
Older adults expect healthcare professionals to ask about their sexual practices and STIs ^*^	17 (37.8%)	19 (42.2%)
Masturbation is only for young people	34 (75.6%)	42 (93.3%)
It is shameful for an older adult to show interest in sex †	34 (75.6%)	41 (91.1%)
I do not believe that older adults also have STIs †	36 (80.0%)	42 (93.3%)
Older adults. after becoming widowed. do not maintain sexual relationships †	23 (51.1%)	39 (87.7%)
Nurses should teach STI prevention to older adults ^*^	44 (97.8%)	45 (100.0%)
Older adults have the right to have sex when. how. and as often as they want ^*^	32 (71.1%)	37 (82.2%)
Older adults do not have sexual activity †	25 (55.6%)	36 (80.0%)
I believe that sex is good for the health of older adults ^*^	35 (77.8%)	39 (86.7%)
I think that older adults with STIs lead a promiscuous life †	23 (51.1%)	32 (71.1%)
It is important to have knowledge about the sexual behavior of older adults ^*^	43 (95.6%)	45 (100.%)
Older adults do not need to use condoms †	38 (84.4%)	42 (93.3%)
Families have the right to interfere with the sexual behavior of older adults †	21(46.7%)	33 (73.3%)
I do not see the need to offer rapid STI testing to older adults †	42 (93.3%)	44 (97.8%)
Affective Component I feel embarrassed when talking to older adults about their sexual behavior †	14 (31.1%)	18 (40.0%)
I feel embarrassed when talking to older adults about STIs †	17 (37.8%)	22 (49.9%)
I trust my technical ability to talk about STIs and their prevention with older adults ^*^	09 (20.0%)	27 (60.0%)
I trust my technical ability to talk about sexual behavior in old age ^*^	09 (20.0%)	28 (62.2%)
I am ashamed to teach an older adult how to use a condom †	18 (40.0%)	18 (40.0%)
Behavioral Component I would allocate time during a nursing consultation to talk about sexual behavior with an older adult ^*^	34 (75.6%)	43 (95.6%)
If an older adult asked me about STIs. I would refer them to another professional †	35(77.8%)	41(91.1%)
During a nursing consultation. I would offer an older adult the opportunity for rapid testing for HIV/syphilis/hepatitis. even if they show no symptoms ^*^	37(82.2%)	44(97.8%)
I would develop health education actions on STI prevention with older adults ^*^	38(84.4%)	43(95.6%)

* *Correct answer - strongly agree; †Correct answer - strongly disagree.*

The assessment of the knowledge and attitude scores of nursing students in Table 3 shows that at the beginning of the study, the average knowledge score was 9.3 points (SD: 1.86) and the average attitude score was 108 points (SD: 11.63). In the post-intervention phase, the average knowledge score was 12.2 points (SD: 0.72) and the average attitude score was 117.2 points (SD: 6.69). These differences in preand post-intervention scores were statistically significant (p<0.001), demonstrating that there was an improvement in the knowledge and attitudes of nursing students about sexual behavior and STIs in older adults after the intervention (Table 3).

**Table 3:** Evaluation of the knowledge and attitude of nursing students about sexual behavior and sexually transmitted infections in older adults, Tangará da Serra, Mato Grosso, Brazil, 2020

Variables		Mean	Median	Standard Deviation	Minimum and Maximum	*p* value
Knowledge	Pre-intervention	09.3	10.0	1.86	05.0 - 13.0	<0.001^*^
	Post-intervention	12.2	12.0	0.72	10.0 - 13.0	
Attitude	Pre-intervention	108.0	109.0	11.63	72.0 - 124.0	<0.001^**^
	Post-intervention	117.2	117.0	6.69	90.0 - 127.0	

* Paired t-test;

** Wilcoxon test.

## DISCUSSION

The relevance of this study lies in the fact that it is the only one that analyzes the knowledge and attitudes of nursing students regarding the sexual behavior and STIs of older adults before and after an educational intervention. Previous quasi-experimental studies have investigated the knowledge and attitudes of nursing students about sexual health and STIs^(19,22,23)^, but not specifically in older adults.

The main finding of this study is that after the intervention, there was a statistically significant increase in the knowledge and attitudes of nursing students regarding sexual behavior and STIs in older adults. Similarly, in the previously cited studies^(19,22,23)^, the increase in students’ knowledge and attitudes was also significant after the intervention.

One explanation for this result is that the students in this study were exposed to an educational intervention whose content was not well known. During their undergraduate studies, theoretical and practical content about sexual behavior and STIs in older adults is not always made available to students. Moreover, as the training data show, most students did not participate in lectures or courses that addressed the topic. This occurs because, generally, sexuality is not considered a priority topic in the training of health professionals, including nursing^(13,24)^.

This deficiency in the training of health professionals results in practices that are also loaded with prejudices, stereotypes, and stigmas. Studies show that they have difficulty addressing sexuality with their patients due to factors such as shame, unpreparedness, lack of confidence, and skills^(9-11,13,25)^.

In health-related courses, content on the sexual health of older adults is limited or nonexistent. In Brazil, professionals in geriatrics and gerontology report that during their undergraduate studies, there is no adequate training on the sexuality of older adults^(14)^. This reality also exists in undergraduate nursing courses^(13,15)^.

The absence of content on the sexuality of people, specifically older adults, in the training of health professionals probably occurs because this topic is rarely addressed in the family, school, and university environments due to being surrounded by taboos, prejudices, and stereotypes^(6,10,24,26)^. Regarding older adults, these stigmas and prejudices are more pronounced. Studies show that health professionals label older adults as asexual and have prejudices about their expression of eroticism or sexual desire^(4-6,11)^.

Another possible explanation for the significant increase in the knowledge and attitudes of the students in this study may be the way the educational intervention was developed. Studies show that educational interventions can be an important strategy for improving knowledge and attitudes regarding sexual health, the sexuality of older adults^(19,23,27)^, and STI prevention^(22)^.

In this research, during the educational intervention, the knowledge and attitudes of students regarding the sexual behavior and STIs of older adults were developed in a differentiated manner. The use of active teaching strategies, such as games, simulations, dynamics, and videos, even remotely through web conferencing, allowed for participation and dialogue among the participants. Additionally, it provided an opportunity to get closer to the reality experienced by older adults, the construction of knowledge, and the development of favorable attitudes towards the addressed topic.

Active teaching strategies are considered pedagogical tools that enhance learning in various areas, including nursing. The student is the active participant, the center of the teaching-learning process^(28)^. Engagement with gerontology content promotes positive attitudes among nursing students towards older adults^(29)^, increases their intention, knowledge, and ability to work with this population^(30)^. Moreover, these strategies can help develop more permissive and less stigmatizing attitudes^(22)^. Attitudes are modified when there is exposure to new information, situations, or experiences^(18)^.

The results of this research demonstrate that the knowledge and attitudes of nursing students regarding sexual behavior and STIs in older adults can significantly improve after an educational intervention. This indicates the necessity for undergraduate nursing programs to include this topic in their curriculum. Furthermore, considering that it is during training that the competencies required for the future performance of nurses are developed, it is crucial that positive attitudes about the sexuality of older adults are encouraged so that they can provide comprehensive and effective care to this population.

### Study Limitations

The sample was composed of nursing students from only one public educational institution. This limits the generalizability of the findings. However, the results point to a reality in nursing courses that is likely similar to that of other institutions. Therefore, it is suggested that similar studies be conducted in other public and private institutions, and that longitudinal studies be developed to investigate the knowledge and attitudes of nursing students about the sexuality and STIs of older adults over time.

### Contributions to Nursing, Health, or Public Policy

The findings of this study can contribute to the professional development of nurses in providing care, as well as in offering comprehensive care to older adults that includes aspects of sexuality. Additionally, these findings can support equitable and comprehensive public policies for promoting healthy aging, focused on STI prevention and providing care centered on the sexuality of older adults. It is worth noting that the contributions of this study can fill gaps in the training of nursing students, thus promoting awareness and sensitization about the importance of equitable and comprehensive health care for older adults, encompassing aspects inherent to the sexual diversity of this population.

## CONCLUSIONS

In this study, after the intervention, the knowledge and attitudes of nursing students regarding sexual behavior and STIs in older adults increased significantly. This is because the educational intervention provided the opportunity to access knowledge not offered during undergraduate studies, in a dynamic and interactive manner.
