# Supplementary material for: Knowledge and attitudes of nursing students regarding the sexuality of older adults: a quasi-experimental study
Source: Rev Bras Enferm. 2024 Dec 13;77(5):e20240011. doi: 10.1590/0034-7167-2024-0011 (PMC11654233; doi:10.1590/0034-7167-2024-0011)
Supplement: Translation

# Translation: Conhecimento e atitudes de estudantes de enfermagem sobre sexualidade de pessoas idosas: estudo quase-experimental

**DOI:** 10.1590/0034-7167-2024-0011pt

**Authors:** Ana Carolina Macri Gaspar Vendramini, Rosemeiry Capriata de Souza Azevedo, Priscila Aguiar Mendes, Janderson Diego Pimenta da Silva, Annelita Almeida Oliveira Reiners, Amanda Cristina de Souza Andrade

**Affiliations:** I Universidade do Estado de Mato Grosso. Tangará da Serra, Mato Grosso, Brasil; II Universidade Federal de Mato Grosso. Cuiabá, Mato Grosso, Brasil; III Universidade Federal de Minas Gerais. Belo Horizonte, Minas Gerais, Brasil

**Keywords:** Pessoa Idosa, Sexualidade, Comportamento Sexual, Estudantes de Enfermagem, Infecções Sexualmente Transmissíveis

## Abstract

**Objetivos:**

comparar o conhecimento e as atitudes de estudantes de enfermagem sobre o comportamento sexual e as Infecções Sexualmente Transmissíveis em pessoas idosas antes e após uma intervenção educativa.

**Métodos:**

estudo quase-experimental, com amostra de conveniência de 45 estudantes de enfermagem de uma universidade pública, realizado em três etapas: pré-intervenção, intervenção e pós-intervenção. Utilizou-se um questionário sobre características sociodemográficas, formação acadêmica e conhecimento e atitudes sobre o tema. A intervenção foi educativa por webconferência. Foram utilizados o teste t pareado e o teste de Wilcoxon na análise dos dados.

**Resultados:**

houve diferença estatisticamente significativa nos escores de conhecimento e atitudes dos estudantes de enfermagem antes e após a intervenção educativa (p < 0,001). Observou-se um aumento significativo no escore de conhecimento (9,3 para 12,2) e atitude (108 para 117,2) na pós-intervenção.

**Conclusões:**

o conhecimento dos estudantes de enfermagem sobre a sexualidade de pessoas idosas aumentou após a intervenção educativa, e suas atitudes relativas à temática tornaram-se mais positivas.

## INTRODUÇÃO

Um dos grandes desafios na atualidade é que, nos últimos anos, em diversos países, como no Brasil, houve um aumento considerável de casos de Infecções Sexualmente Transmissíveis (IST) em pessoas mais velhas^(1-4)^. Dados epidemiológicos mostram que no Japão, um país de alta renda, por exemplo, os casos de sífilis aumentaram cerca de 8 vezes, de 165 em 2009 para 1.280 em 2019, com um aumento de 28,7% em homens e 23,1% em mulheres da taxa de incidência em adultos mais velhos^(1)^. Já no Brasil, um país de renda média alta, com relação ao HIV, houve uma tendência de estabilidade, porém, com aumento da incidência em pessoas idosas com 60 anos ou mais, passando de 4,25/100.000 habitantes em 2007 para 8,73/100.000 habitantes em 2020, na região nordeste^(2)^.

Diversos fatores relacionados ao comportamento sexual de pessoas idosas têm contribuído para sua vulnerabilidade às IST, como a baixa percepção de risco para contrair IST^(5)^ e a prática sexual desprotegida^(4,6)^. Além disso, elas possuem pouco conhecimento sobre métodos de prevenção de IST e Aids^(5,6)^ e não procuram as unidades de saúde para realizar a testagem para IST/HIV^(7)^, contribuindo para um possível diagnóstico de HIV tardio.

Outros fatores relacionam-se aos profissionais de saúde, sobre os quais a literatura indica terem dificuldades em abordar a temática da sexualidade durante o atendimento da população idosa^(8,9)^. A maioria dos profissionais, inclusive enfermeiros, possui estigmas e estereótipos em relação à sexualidade de pessoas idosas, por acreditar que nessa fase da vida não há interesse sexual e não há vida sexual ativa^(9-11)^. Eles sentem-se constrangidos ao falar sobre o assunto e têm atitudes negativas em relação à atividade ou desejo sexual dessa população^(12)^.

Ademais, eles possuem pouco conhecimento acerca do HIV/Aids em pessoas idosas^(7)^, assim como sobre seu comportamento sexual^(10)^. Um dos motivos para que isso aconteça é que a formação acadêmica desses profissionais da saúde é insuficiente e/ou inexistente quanto a conteúdos sobre sexualidade no envelhecer^(11,13-15)^.

Na enfermagem, poucas pesquisas investigaram o conhecimento e a atitude de estudantes sobre sexualidade, comportamento sexual ou IST específicas da população idosa. Entretanto, aquelas existentes apontam para um conhecimento insatisfatório sobre o assunto e as atitudes oscilam entre positivas e negativas^(16,17)^. Conhecimento pode ser considerado como o que a pessoa sabe/conhece sobre o fenômeno. A atitude é uma reação psicológica expressa por uma avaliação positiva ou negativa, favorável ou desfavorável de um objeto, pessoa ou acontecimento e possui componentes cognitivos (informações), afetivos (sentimentos) e comportamentais (intenções para ação)^(18)^.

Pesquisas têm demonstrado que o nível de conhecimento de estudantes sobre saúde sexual pode ser melhorado com programas de intervenção educativa, bem como promoção de mudanças de atitudes^(7,19)^, mas esses estudos são escassos. Ademais, nenhum destes aborda saúde sexual e IST especificamente de pessoas idosas^(7,19)^. Diante desse contexto, essa pesquisa parte da hipótese de que uma intervenção educativa é capaz de melhorar o conhecimento e as atitudes de estudantes de enfermagem sobre o comportamento sexual e IST em pessoas idosas.

## OBJETIVOS

Comparar o conhecimento e as atitudes de estudantes de enfermagem sobre o comportamento sexual e as IST em pessoas idosas antes e após uma intervenção educativa.

## MÉTODOS

### Aspectos éticos

A pesquisa foi aprovada pelo Comitê de Ética em Pesquisa (CEP), e nos Registros Brasileiros de Ensaios Clínicos (REBEC) sob o número RBR-3rkg96p.

### Desenho, período e local do estudo

Estudo desenvolvido no período de 2020 a 2021, em uma universidade pública do estado de Mato Grosso. Trata-se de uma pesquisa quase-experimental, do tipo antes e depois, com grupo intervenção, norteada pela ferramenta CONSORT.

### População, critérios de inclusão e exclusão

A população do estudo foi composta por 62 estudantes matriculados no curso de enfermagem da referida universidade, que cursavam a partir do sétimo semestre até o décimo. Esse critério foi utilizado em virtude da disciplina de saúde do idoso ser ofertada no sétimo semestre. Foram excluídos aqueles que não frequentavam as aulas mesmo estando matriculados.

A amostra foi estabelecida antes da coleta de dados a partir do cálculo amostral para medidas repetidas, considerando 5% de nível de significância, poder de 80% e diferença mínima antes e após a intervenção de 2,5 unidades, conforme um estudo^(19)^, totalizando 32 indivíduos. Ao calcular um percentual de 25% de perda, a amostra mínima seria de 43 estudantes. Estabeleceram-se os seguintes critérios de inclusão: ser acadêmico com idade igual ou superior a 18 anos e de ambos os sexos.

Todos os 62 estudantes matriculados foram convidados para participar da pesquisa. Dentre eles, 54 aceitaram o convite. Oito estudantes foram descontinuados do estudo, pois não concluíram todas as etapas da pesquisa: seis não participaram do primeiro encontro da intervenção e dois do segundo. Ao final, a amostra foi composta por 45 estudantes de enfermagem.

### Protocolo do estudo

A coleta de dados aconteceu nos meses de novembro e dezembro de 2020, em três etapas: pré-intervenção, intervenção e pós-intervenção, conforme Figura 1.

**Figura 1:** Representação do fluxograma de coleta de dados e programa de intervenção

*ATIV - Atividade; †AC - Acolhimento; ǂ*DIN - Dinâmica; V/F - Verdadeiro/Falso;* ǀǀ*VID - Vídeo; ¶ROT - Roteiro; §ROL - Roleta.*

Na fase pré-intervenção (T1), realizou-se o recrutamento dos estudantes de enfermagem. O convite foi feito por meio de mensagens em grupo de WhatsApp^®^ e e-mails contendo carta-convite com informações da pesquisa, Termo de Consentimento Livre e Esclarecido (TCLE) assinado pela pesquisadora e link de acesso ao questionário da pesquisa via plataforma do *Google Forms* ^®^.

Na segunda fase, a intervenção, os 54 estudantes foram distribuídos em quatro grupos de acordo com o semestre no qual estavam matriculados. Com cada grupo, realizaram-se dois encontros agendados previamente, em dias subsequentes, com duração de 2 horas. Cada encontro (T2 e T3) foi planejado com uma temática específica. O primeiro, destinado a abordar atividades sobre o comportamento sexual de pessoas idosas, e o segundo, sobre IST nessa população.

A intervenção foi educativa, realizada on-line (em virtude do período de pandemia da covid-19), por webconferência, com o aplicativo de reunião *Google Meet* ^®^, utilizando a técnica de oficinas, que permite um espaço dialógico no processo de ensino-aprendizagem. As atividades foram desenvolvidas pela pesquisadora e acompanhadas por um tutor, que se responsabilizava pela organização das atividades e registro de frequência dos participantes. Durante todas as atividades, estabeleceu-se que os participantes deveriam permanecer com as câmeras ligadas.

No primeiro encontro (T2), realizaram-se o acolhimento dos participantes, a socialização, a informação sobre as atividades a serem desenvolvidas e a checagem da frequência; em seguida, desenvolveu-se uma dinâmica com a finalidade de estimular os estudantes a expressar suas ideias sobre o tema. Para tal, utilizou-se o método de associação livre de ideias e o recurso do quadro digital interativo, a partir da seguinte pergunta: “O que lhe vem à cabeça quando ouve a frase ‘sexualidade de pessoas idosas’?”. À medida que os estudantes expressavam as palavras, elas eram registradas no quadro.

Na sequência, aplicou-se uma dinâmica denominada Verdadeiro ou Falso com a finalidade de melhorar o conhecimento e desmistificar mitos dos estudantes sobre o comportamento sexual de pessoas idosas. A pesquisadora apresentava aos participantes frases que abordavam o comportamento sexual de pessoas idosas e eles deveriam responder: verdadeiro, falso ou não sei; em seguida, a resposta correta foi apresentada pela pesquisadora com base em evidências científicas.

Outra atividade desenvolvida foi a apresentação de um vídeo sobre sexualidade de pessoas idosas, disponível em uma plataforma on-line^(20)^, cuja finalidade foi melhorar as atitudes dos estudantes sobre o comportamento sexual de pessoas idosas. Ao término do vídeo, destinou-se um espaço para debate acerca das informações e percepções apreendidas no vídeo. Ao final desse encontro, solicitou-se aos participantes que elaborassem um roteiro, a ser apresentado no início do próximo encontro, que abordasse o comportamento sexual das pessoas idosas durante uma consulta de enfermagem.

No segundo dia da intervenção (T3), discutiu-se o roteiro previamente elaborado pelos participantes e, ao final, um único instrumento foi construído e socializado para todos. Essa atividade teve como finalidade trabalhar a abordagem sobre sexualidade de pessoas idosas, realizada pelos estudantes durante uma consulta de enfermagem.

Dando continuidade, a fim de melhorar o conhecimento dos estudantes sobre as IST, apresentou-se um vídeo, produzido pela pesquisadora, com informações epidemiológicas sobre IST e políticas de prevenção dessas doenças no Brasil e no mundo. Na sequência, visando melhorar as atitudes dos estudantes em relação a pessoas idosas com IST, apresentou-se outro vídeo com depoimentos de pessoas idosas que vivem com HIV/Aids, disponível em plataforma on-line^(21)^. Após a apresentação de cada vídeo, houve um espaço para diálogo sobre o assunto entre os participantes.

Posteriormente, com a finalidade de melhorar as atitudes dos estudantes em relação às pessoas idosas com IST, realizou-se uma dinâmica denominada pela pesquisadora como roleta virtual, na qual havia números referentes a perguntas elaboradas pela pesquisadora sobre as ações de profissionais de saúde relacionadas à promoção da saúde sexual e prevenção de IST em pessoas idosas. Cada acadêmico deveria responder à pergunta referente ao número selecionado na roleta. Após as respostas dos participantes, a pesquisadora complementava apresentando resultados de estudos sobre a temática, abrindo espaços para reflexões e debates no grupo.

Para concluir a atividade educativa, a pesquisadora retomou a dinâmica realizada no primeiro encontro, na qual foi feita a pergunta sobre a sexualidade de pessoas idosas; em seguida, apresentou-se o resultado dos dois quadros produzidos aos estudantes, que foram estimulados a comparar as mudanças ocorridas após a participação na intervenção. A pesquisadora finalizou a atividade dando oportunidade para que os estudantes avaliassem a intervenção.

Ainda no segundo encontro, realizou-se a pós-intervenção (T4). Para tal, aplicou-se o mesmo instrumento sobre o conhecimento e a atitude dos estudantes de enfermagem sobre comportamento sexual e IST de pessoas idosas utilizado na fase de pré-intervenção.

### Variáveis de estudo

As variáveis do estudo foram as características sociodemográficas (sexo, faixa etária, estado civil, religião, contato próximo com pessoas idosas, residir com pessoas idosas); formação acadêmica (semestre em curso, conteúdo sobre comportamento sexual de pessoas idosas, conteúdo sobre IST em pessoas idosas, atividade complementar sobre comportamento sexual e IST, pesquisa com pessoas idosas, extensão com pessoas idosas, prática assistencial com pessoas idosas); conhecimentos e atitudes sobre o comportamento sexual e as IST em pessoas idosas (média de conhecimento e atitude).

### Instrumento de coleta de dados

Os dados foram coletados por meio de um questionário para identificar as características dos participantes do estudo. Este compreendeu perguntas fechadas acerca dos aspectos sociodemográficos e de formação dos estudantes.

Para identificar o conhecimento e atitude, utilizou-se uma escala criada pelos pesquisadores acerca do conhecimento e atitudes sobre comportamento sexual e IST em pessoas idosas, que passou por processo de validação de conteúdo por um comitê de oito juízes com expertise na área, cujo índice de validade de conteúdo (IVC) foi de 96%.

A escala do conhecimento é constituída de 13 perguntas com respostas do tipo: verdadeiro, falso e não sei. Para cada resposta correta, considerou-se um ponto de corte e, ao final, seria possível obter um escore entre 0 e 13 pontos. Quanto às atitudes, a escala possui 26 questões com respostas do tipo Likert, variando entre: concordo totalmente, concordo parcialmente, indeciso, discordo parcialmente e discordo totalmente. O escore final varia de 1 a 130 pontos. Determinou-se que quanto maior o escore, melhor o conhecimento e mais positivas são as atitudes.

### Análise dos resultados e estatística

Os dados foram organizados em um banco de dados com auxílio do programa estatístico Stata, versão 16. Para analisar os dados, realizaram-se frequências absoluta e relativa, média, mediana, valor mínimo, máximo e desvio-padrão das variáveis. Para identificar as mudanças no conhecimento e atitude, realizou-se avaliação dos resultados obtidos por meio da aplicação do instrumento antes e após a intervenção. Inicialmente, testou-se a normalidade dos dados utilizando o teste de Shapiro-Wilk. Para comparação dos escores de conhecimento e atitude dos estudantes de enfermagem sobre comportamento sexual e IST em pessoas idosas antes e após a intervenção, utilizou-se o teste t pareado ou de Wilcoxon, sendo estabelecido nível de significância de 5%.

## RESULTADOS

Dos 45 estudantes de enfermagem, 93,3% são do sexo feminino, têm média de idade de 25 anos, 60% são solteiros e 46,5% são protestantes. A maioria (75,6%) não reside com pessoas acima de 60 anos. Dos que referiram (83,6%) ter contato próximo com pessoas idosas, 35,6% têm contato com avós, 22,5% com pais e/ou tios e 22,5% com pais e/ou avós.

Com relação à formação acadêmica, 26,7% dos estudantes cursam o sétimo e oitavo semestres. Durante o curso, a maior parte (55,6%) obteve algum conteúdo sobre comportamento sexual de pessoas idosas, porém 53,3% não obtiveram conteúdo significativo sobre IST nessa população. Respectivamente, 93,3% e 91,1% dos estudantes não participaram de palestras sobre comportamento sexual ou IST em pessoas idosas. Pouco mais de 60% dos estudantes realizaram prática assistencial com pessoas idosas sem abordar aspectos do comportamento sexual ou IST. Com relação à pesquisa e extensão envolvendo a população idosa, a maioria não participou de ambas as atividades (86,7% e 82,2%, respectivamente). Quase metade dos estudantes (44,4%) autorreferiram ter pouco conhecimento sobre comportamento sexual de pessoas idosas e IST.

Observa-se na Tabela 1 que os resultados relacionados ao conhecimento dos estudantes de enfermagem sobre o comportamento sexual e as IST em pessoas idosas mostram que houve maior porcentagem de respostas corretas na pós-intervenção quando comparada à da pré-intervenção. Destacam-se, sobre o comportamento sexual, as questões que tratam sobre relacionamentos casuais, uso de brinquedos sexuais e filmes para melhorar o desempenho sexual e uso do preservativo. Sobre as IST, a pergunta acerca das políticas disponíveis no Brasil sobre prevenção de IST na população idosa foi a que apresentou maior mudança na porcentagem de acerto.

**Tabela 1:** Distribuição dos estudantes de enfermagem segundo itens da escala de conhecimento sobre comportamento sexual e infecções sexualmente transmissíveis em pessoas idosas pré e pós-intervenção, Tangará da Serra, Mato Grosso, Brasil, 2020

Variáveis	Pré-intervenção			Pós-intervenção		
	Falso n(%)	Verdadeiro n(%)	Não sei n(%)	Falso n(%)	Verdadeiro n(%)	Não sei n(%)
Pessoa idosas não têm vida sexual ativa	37(82,2%)	8 (17,8%)		42(93,3%)	3 (6,7%)	
Processo de envelhecimento provoca mudanças fisiológicas que influenciam no desempenho sexual das pessoas idosas	-	45(100%)	-	-	45(100%)	-
O desejo sexual permanece na velhice	1(2,2%)	43(95,6%)	1(2,2%)	-	45(100%)	-
Mulheres idosas avaliam sua satisfação sexual de maneira mais positiva que os homens idosos	23(51,1%)	8(17,8%)	14 (31,1%)	31(68,9%)	10(22,2%)	4(8,9%)
Relações sexuais casuais fazem parte do comportamento sexual de pessoas idosas	7(15,6%)	27(60%)	11(24,4%)	-	45(100%)	-
A frequência da atividade sexual de pessoas idosas diminui com a idade	10(22,2%)	30(66,7%)	5(11,1%)	11(24,4%)	34(75,56%)	-
A atividade sexual para pessoas idosas não se restringe apenas à relação sexual, pode ser beijar, abraçar, fazer carícias e masturbação	-	43(95,6%)	2(4,4%)	-	45(100%)	-
Pessoas idosas utilizam brinquedos sexuais ou filmes para melhorar o desempenho sexual	2(4,4%)	20(44,4%)	23(51,2%)	-	44(97,8%)	1(2,2%)
Pessoas idosas utilizam medicamentos para melhorar o desempenho sexual	-	41(91,1%)	4(8,9%)	-	45(100%)	-
Pessoas idosas costumam utilizar o preservativos nas relações sexuais	30(66,7%)	4 (8,9%)	11(24,4%)	41(91,1%)	3 (6,7%)	1(2,2%)
Há muitos casos de HIV/Aids, hepatites e sífilis na população idosa	-	36(80,0%)	9 (20,0%)	-	45(100%)	-
O diagnóstico de HIV/Aids em pessoas idosas é tardio	1(2,2%)	35 (77,8%)	9 (20,0%)	-	45(100%)	-
No Brasil, há uma política para prevenção de IST/HIV/Aids em pessoas idosas	11(24,4%)	9(20,0%)	25(55,6%)	40(88,9%)	5(11,1%)	-

Quanto às atitudes dos estudantes de enfermagem sobre o comportamento sexual e as IST em pessoas idosas apresentadas na Tabela 2, percebe-se mudanças em todos os componentes da atitude, cognitivos, afetivos e comportamentais, após a intervenção. A porcentagem de respostas corretas de estudantes na pós-intervenção foi maior para todas as perguntas, exceto uma (sentimento de vergonha para ensinar a pessoa idosa a utilizar a camisinha).

**Tabela 2:** Distribuição dos estudantes de enfermagem segundo escala de atitude sobre comportamento sexual e infecções sexualmente transmissíveis em pessoas idosas pré e pós-intervenção, Tangará da Serra, Mato Grosso, Brasil, 2020

Variáveis	Pré-intervenção Resposta correta n(%)	Pós-intervenção Resposta correta n(%)
Componente cognitivo		
Considero ser importante avaliar comportamento sexual de pessoas idosas na consulta de enfermagem ^^*^^	44 (97,8%)	45 (100,0%)
Acredito que a maioria das pessoas idosas deixa de se interessar por sexo†	02 (4,4%)	27(60,0%)
Profissionais de saúde esperam que as pessoas idosas perguntem sobre prática sexual e IST †	05 (11,1%)	12 (26,7%)
Pessoas idosas esperam que os profissionais de saúde perguntem sobre sua prática sexual e IST ^*^	17 (37,8%)	19 (42,2%)
Masturbação é apenas para jovens	34 (75,6%)	42 (93,3%)
É vergonhoso uma pessoa idosa demonstrar interesse por sexo†	34 (75,6%)	41 (91,1%)
Não acredito que pessoas idosas também têm IST †	36 (80,0%)	42 (93,3%)
Pessoas idosas, após ficarem viúvos, não mantêm relacionamento sexual †	23 (51,1%)	39 (87,7%)
O enfermeiro deve ensinar a prevenção das IST às pessoas idosas^*^	44 (97,8%)	45 (100,0%)
Pessoas idosas têm direito de fazer sexo quando, quanto e como quiserem^*^	32 (71,1%)	37 (82,2%)
Pessoa idosas mais velhas não têm atividade sexual †	25 (55,6%)	36 (80,0%)
Acredito que o sexo faz bem para saúde das pessoas idosas ^^*^^	35 (77,8%)	39 (86,7%)
Penso que pessoas idosas com IST têm vida promíscua†	23 (51,1%)	32 (71,1%)
É importante ter conhecimento sobre comportamento sexual de pessoas idosas ^^*^^	43 (95,6%)	45 (100,%)
Pessoas idosas não precisam usar o preservativo†	38 (84,4%)	42 (93,3%)
A família tem o direito de interferir no comportamento sexual das pessoas idosas†	21(46,7%)	33 (73,3%)
Não vejo necessidade de oferecer o teste rápido para IST para as pessoas idosas †	42 (93,3%)	44 (97,8%)
Componente afetivo Sinto-me constrangido ao conversar com pessoas idosas sobre seu comportamento sexual†	14 (31,1%)	18 (40,0%)
Sinto-me constrangido ao conversar com pessoas idosas sobre IST†	17 (37,8%)	22 (49,9%)
Confio na minha capacidade técnica para falar sobre IST e sua prevenção com pessoas idosas ^^*^^	09 (20,0%)	27 (60,0%)
Confio na minha capacidade técnica para falar sobre comportamento sexual na velhice^^*^^	09 (20,0%)	28 (62,2%)
Tenho vergonha de ensinar uma pessoa idosa a utilizar o preservativo†	18 (40,0%)	18 (40,0%)
Componente comportamental Reservaria um tempo da consulta de enfermagem para falar sobre comportamento sexual com pessoa idosa^^*^^	34 (75,6%)	43 (95,6%)
Se alguma pessoa idosa me questionasse sobre IST, eu o encaminharia para outro profissional†	35(77,8%)	41(91,1%)
Durante uma consulta de enfermagem, eu ofereceria para pessoa idosa a oportunidade de realizar testagem rápida para HIV/sífilis/hepatite, mesmo que não apresente sintomas ^^*^^	37(82,2%)	44(97,8%)
Desenvolveria ações de educação em saúde sobre prevenção de IST com pessoas idosas^*^	38(84,4%)	43(95,6%)

* *Resposta correta - concordo totalmente; †Resposta correta - discordo totalmente.*

A avaliação do escore de conhecimento e atitude dos estudantes de enfermagem na Tabela 3, mostra que no início do estudo a média de acerto dos itens de conhecimento era de 9,3 pontos (DP: 1,86) e da atitude a média do escore foi de 108 pontos (DP: 11,63). Na fase pós-intervenção, a média do conhecimento foi de 12,2 pontos (DP: 0,72) e da atitude de 117,2 pontos (DP: 6.69). Essas diferenças nos escores pré e pós-intervenção foram estatisticamente significativas (p<0,001), demonstrando que houve melhora no conhecimento e nas atitudes dos estudantes de enfermagem sobre comportamento sexual e IST em pessoas idosas após a intervenção (Tabela 3).

**Tabela 3:** Avaliação do conhecimento e da atitude dos estudantes de enfermagem sobre comportamento sexual e infecções sexualmente transmissíveis em pessoas idosas, Tangará da Serra, Mato Grosso, Brasil, 2020

Variáveis		Média	Mediana	Desvio- Padrão	Mínimo e Máximo	Valor de *p*
Conhecimento	Pré	09,3	10,0	01,86	05,0 - 13,0	<0,001^*^
	Pós	12,2	12,0	00,72	10,0 - 13,0	
Atitude	Pré	108,0	109,0	11,63	72,0 - 124,0	<0,001^**^
	Pós	117,2	117,0	006,69	90,0 - 127,0	

* Teste T-Pareado;

** Teste Wilcoxon.

## DISCUSSÃO

A relevância deste estudo reside no fato de que é o único que analisa o conhecimento e as atitudes de estudantes de enfermagem sobre o comportamento sexual e IST das pessoas idosas, antes e após uma intervenção educativa. Estudos anteriores, com desenho quase-experimental, investigaram conhecimentos e atitudes de estudantes de enfermagem sobre saúde sexual e IST^(19,22,23)^, mas não especificamente de pessoas idosas.

O principal achado deste estudo é que, depois da intervenção, houve um aumento estatisticamente significativo do conhecimento e das atitudes dos estudantes de enfermagem sobre comportamento sexual e IST em pessoas idosas. De maneira semelhante, nos estudos anteriormente citados^(19,22,23)^, após a intervenção, o aumento do conhecimento e das atitudes dos estudantes também foi significativo.

O que pode explicar esse resultado é que os estudantes deste estudo foram expostos a uma intervenção educativa cujo conteúdo era pouco conhecido. Durante a graduação, o conteúdo teórico e prático sobre comportamento sexual e IST em pessoas idosas nem sempre é oportunizado aos estudantes. Além disso, como os dados de formação apontam, a maioria dos estudantes não participou de palestras ou cursos que abordassem a temática. Isso acontece porque, de modo geral, a sexualidade é uma temática não considerada como prioritária na formação dos profissionais de saúde, incluindo os de enfermagem^(13,24)^.

Essa deficiência na formação dos profissionais de saúde resulta em práticas também carregadas de preconceitos, estereótipos e estigmas. Estudos mostram que eles têm dificuldade de abordar a sexualidade com seus pacientes devido a fatores como vergonha, despreparo, falta de confiança e habilidades^(9-11,13,25)^.

Nos cursos da área da saúde, o conteúdo sobre saúde sexual de pessoas idosas é limitado ou inexistente. No Brasil, profissionais da área de geriatria e gerontologia referem que na graduação não há formação adequada sobre a sexualidade de pessoas idosas^(14)^. Nos cursos de graduação em Enfermagem, essa realidade também existe^(13,15)^.

Provavelmente, a ausência do conteúdo da sexualidade das pessoas e, em específico, de pessoas idosas na formação dos profissionais de saúde acontece devido a esse tema ser pouco abordado no meio familiar, escolar e também nas universidades, por ser cercado por tabus, preconceitos e estereótipos^(6,10,24,26)^. Em relação às pessoas idosas, esses estigmas e preconceitos são mais acentuados. Estudos mostram que os profissionais da saúde rotulam as pessoas idosas como assexuadas e têm preconceitos quanto à sua expressão de erotismo ou desejo sexual^(4-6,11)^.

Outra possível explicação para o aumento significativo do conhecimento e das atitudes dos estudantes deste estudo pode ser devido à forma como a intervenção educativa foi desenvolvida. Estudos mostram que intervenções educativas podem ser uma importante estratégia para melhorar conhecimentos e atitudes em relação à saúde sexual, sexualidade de pessoas idosas^(19,23,27)^ e prevenção de IST^(22)^.

Nesta pesquisa, na intervenção educativa, o conhecimento e as atitudes dos estudantes em relação ao comportamento sexual e IST de pessoas idosas foram desenvolvidos de maneira diferenciada. O uso de estratégias de ensino ativas, como jogos, simulação, dinâmicas e vídeos, ainda que de forma remota, por webconferência, permitiu a participação e o diálogo entre os participantes. Além disso, oportunizou sua aproximação com a realidade vivenciada pelas pessoas idosas, a construção de conhecimentos e o desenvolvimento de atitudes favoráveis em relação ao assunto abordado.

As estratégias de ensino ativas são consideradas instrumentos pedagógicos que potencializam a aprendizagem em diversas áreas, inclusive na enfermagem. O aluno é o participante ativo, o centro do processo de ensino-aprendizagem^(28)^. O envolvimento com o conteúdo de gerontologia promove atitudes positivas de estudantes de enfermagem em relação às pessoas idosas^(29)^, aumenta a intenção, o conhecimento e a capacidade em trabalhar com essa população^(30)^. Além disso, essas estratégias podem auxiliar no desenvolvimento de atitudes mais permissivas e menos estigmatizantes^(22)^. Atitudes são modificadas quando há exposição a novas informações, situações ou experiências^(18)^.

Os resultados desta pesquisa demonstram que o conhecimento e as atitudes de estudantes de enfermagem sobre comportamento sexual e IST em pessoas idosas podem melhorar, significativamente, após uma intervenção educativa. Isso mostra a necessidade de os cursos de graduação em Enfermagem incluírem essa temática no processo de formação dos estudantes. Ademais, tendo em vista que é durante a formação que se desenvolvem as competências necessárias para o desempenho das funções dos enfermeiros no futuro, é relevante que atitudes positivas acerca da sexualidade de pessoas idosas sejam estimuladas para que eles possam atender essa população de maneira integral e efetiva.

### Limitações do estudo

A amostra foi composta por estudantes de um curso de enfermagem de apenas uma instituição pública de ensino. Isso dificulta a generalização dos achados. Porém, os resultados encontrados apontam para uma realidade dos cursos de enfermagem que provavelmente é similar à de outras instituições. Nesse sentido, sugere-se a realização de estudos semelhantes em outras instituições públicas e privadas, e que estudos longitudinais sejam desenvolvidos para investigar o conhecimento e as atitudes dos estudantes de enfermagem sobre sexualidade e IST de pessoas idosas ao longo do tempo.

### Contribuições para a área da enfermagem, saúde ou política pública

Os achados deste estudo podem contribuir para o aprimoramento profissional do(a) enfermeiro(a) na prestação de cuidados, bem como na oferta de uma assistência integral à pessoa idosa, que compreenda aspectos inerentes à sexualidade. Além disso, esses achados podem subsidiar políticas públicas equitativas e integrais para promoção do envelhecimento saudável, focadas na prevenção de IST e na prestação de cuidados centrados na sexualidade de pessoas idosas. Vale ressaltar que as contribuições deste estudo podem preencher lacunas no processo de formação dos estudantes de enfermagem, promovendo assim a conscientização e sensibilização sobre a importância de uma assistência equânime e integral à saúde das pessoas idosas, contemplando aspectos inerentes à diversidade sexual dessa população.

## CONCLUSÕES

Neste estudo, após a intervenção, o conhecimento e as atitudes dos estudantes de enfermagem sobre o comportamento sexual e IST em pessoas idosas aumentaram significativamente. Isso porque a intervenção educativa possibilitou a oportunidade de acessar um conhecimento não ofertado durante a graduação, de modo dinâmico e interativo.
